# A review of the prescribing culture of anti-depressants across government districts in Northern Ireland

**DOI:** 10.3389/fdsfr.2023.1303572

**Published:** 2023-12-21

**Authors:** Mark W. Ruddock, Joanne Watt, Mary Jo Kurth, John V. Lamont, Laura Mooney, Peter Fitzgerald

**Affiliations:** ^1^ Randox Laboratories Ltd., Clinical Studies Group, Randox Science Park, Antrim, United Kingdom; ^2^ Randox Health, Crumlin, United Kingdom

**Keywords:** anti-depressant, anti-anxiety, Northern Ireland, prescriptions, medications, mental health, depression, citalopram

## Abstract

**Introduction:** The COVID-19 pandemic has caused a significant increase in mental health issues which general practitioners are now witnessing and managing in communities across Northern Ireland. Unfortunately, this new tsunami of patients with mental health issues has put tremendous strain on our already overburdened health system. As a result, Northern Ireland currently holds the unenviable record for prescribing more anti-anxiety and anti-depressant medication than any other country in the world.

**Methods:** Data was obtained from the Northern Ireland Statistics and Research Agency (NISRA), Family Practitioner Services, General Pharmaceutical Services, Annual Statistics 2020/2021 (published June 2021) and 2021/2022 (published June 2022). Data was analysed by age, gender, district, and socioeconomic class on prescription medication [according to the British National Formulary (BNF)].

**Results:** From 2020/2021 to 2021/2022, the prescribing culture for anti-anxiety and/or anti-depressant medication in Northern Ireland did not abate (24% vs. 14%, female to male, respectively). The postcode and index of multiple deprivation (IMD) was analysed and a mean IMD for each constituency was taken as an estimate of the overall IMD to establish if money spent per patient was related to the IMD in each constituency. North Down, South Antrim, and East Antrim were least deprived, as indicated by their high IMD. Whereas, Foyle, and Belfast West were most deprived (low IMD). The cost of mood and anxiety medication per patient was compared against constituency; patients in Belfast West and Belfast North, followed by Foyle, had the highest costs per patient, and the lowest IMD (most deprived).

**Conclusion:** This review concludes that there has been no change in the prescribing culture for anti-anxiety or anti-depressants across Northern Ireland (2020–2022). The cost of mood and anxiety medication per patient did not correlate with the index of multiple deprivation (IMD). Areas of low IMD trended to have higher spend. Is it now time to review the prescribing culture in Northern Ireland and offer greater support to our GPs to initiate a program of deprescribing and manage the wellbeing of our citizens?

## Introduction

One of the primary reasons for the increase in the prescribing culture for anti-anxiety and anti-depressant medication (s) in Northern Ireland is due to the lack of access to mental health services e.g., access to mental health professionals and associated alternative therapies (e.g., counselling, cognitive behaviour therapy (CBT), behavioural activation, interpersonal therapy (IPT), eye movement desensitisation and reprocessing (EMDR), mindfulness-based cognitive therapy (MBCT), psychodynamic psychotherapy, and couple therapy).

In Northern Ireland, the Participation and the Practice of Rights (PPR) group highlighted concerns that a “postcode lottery” operates for people who need access to counselling. In addition, general practitioners (GPs) have a limited time with their patients (on average <7–10 min/patient) and waiting lists continue to grow. To bridge this gap, GPs have continued to prescribe anti-anxiety and/or anti-depressant medications, not knowing if the drug will benefit the patient or not. This should be a concern to all of us as this prevailing prescription culture is now a serious threat to our health, and the long-term use of anti-anxiety and/or anti-depressant medication, and subsequent withdrawal, is currently unknown.

Anti-anxiety and anti-depressant medications are two of the most commonly prescribed drugs in the United Kingdom (UK) (Iacobucci, 2019). A normal course of anti-depressants should last at least 6 months (Anderson et al., 2008). However, in patients at potential risk of relapse, this can be at least 2 years, or longer (Anderson et al., 2008). In Northern Ireland almost 20% of the population were prescribed an anti-anxiety and/or anti-depressant medication during 2020/2021 (Department of Health, 2023). Currently, 24% of females and 13.8% of males in Northern Ireland are taking an anti-anxiety and/or anti-depressant medication (s) (Department of Health, 2023). However, in some of the most deprived areas within Northern Ireland, the number of prescription items dispensed are almost 50% higher (Department of Health, 2023). Overall, GPs in Northern Ireland prescribed enough anti-depressant medication to give each member of the population a 27-day supply, as compared to 10-day supply in England and a 19-day supply in Wales (The Irish Times, 2023). In just 15 years, the number of prescriptions written by GPs across the UK has increased three-fold (GOV.UK, 2023). As a result, Northern Ireland holds one of the world’s unenviable records for the highest prescription rates for anti-depressant medications (BBC News, 2023b).

Surprisingly, in Northern Ireland there are currently limited analyses of the socioeconomic context for this pattern of prescribing (Frazer and Frazer, 2020). Moreover, the Office for Statistics Regulation [Review of Mental Health Statistics in Northern Ireland (September 2021)] have reported that there is (a) a scarcity of robust mental health data in Northern Ireland, (b) a lack of official statistics, (c) no accurate regional picture of mental health, and (d) there is no single point of access to official statistics on mental health (Office for Statistics Regulation, 2023).

Although poverty and the legacy of the Troubles in Northern Ireland has been identified as a potential driver for why so many patients are prescribed anti-anxiety and/or anti-depressant medications, doctors have indicated that new patients are too young to have been directly affected. However, there is the suggestion that the era of social media is a significant contributing factor to the mental health problems of many young people—in particular, females (Naslund et al., 2020; Lupton, 2021; Rifkin-Zybutz et al., 2023). Furthermore, a recent report published by the University of Pittsburgh suggested that the more time that a user invested on social media [Social Media Addiction (SMA)], the more likely they were predisposed to mental health issues e.g., anxiety and depression (Shensa et al., 2017). Typical signs and symptoms of SMA include: (i) spending too much time on social media, thinking about social media, and/or creating new social media content, (ii) fixating on “likes” and “followers,” (iii) comparing daily life with others with a constant need to impress, (iv) neglecting offline relationships, (v) a lack of focus on things other than social media, (vi) a feeling of restlessness, anxious, or agitated when unable to access social media, (vii) using social media to escape reality, (viii) and increasing social media use over time to achieve the same gratification i.e., SMA (Sun and Zhang, 2021; Thomson et al., 2021; Xiao et al., 2022). The consequences and effects of SMA include (but are not limited to), (a) procrastination, (b) poor time management, (c) reduced work and/or academic performance, (d) poor mental health, (e) lack of fulfilment, (f) decreased physical activity (sedentary lifestyle), (g) social anxiety, (h) disrupted sleep patterns (problems falling asleep and problems staying asleep), (i) reduced connection to people in “real life,” and (j) depression (Karim et al., 2020; Alonzo et al., 2021).

In a recent study conducted by the National Health Service (NHS) and AWARE (depression charity for Northern Ireland), it was reported that females in Northern Ireland were more likely to show signs of mental health problems when compared to males (Aware Ni, 2023). Mental health illness in females has also soared in England (NHS England, 2023). Alarmingly, the NHS-based study reported that 12.6% of the female population, aged between 16–24, screened positive for post-traumatic stress disorder (PTSD) (females are twice as likely to screen positive for PTSD than males; 10% vs. 4%, respectively); 19.7% of the females that screened positive for PTSD also reported that they self-harmed, and over 28% had other mental health issues (The Guardian, 2023). Therefore, it is not surprising that the number of prescriptions for anti-anxiety and/or anti-depressant medications have continued to increase year on year throughout the UK.

In 2020, the total spent on anti-depressant drugs in Northern Ireland increased by almost £7 million, in just 1 year (£11.3 million in 2019 to £18.3 million in 2020), an increase of 38.3% (BBC News, 2023b). Sertraline (50 mg), one of the most commonly prescribed anti-depressant drugs used to treat symptoms of anxiety and depression, increased in price (raw material costs) from £1.27 for a pack of 28 tablets in January 2020, to £6.46 in May 2020; a 5-fold increase in the cost of these medications. Albeit, this anomaly was due to the COVID-19 pandemic. However, it demonstrated that in any future pandemic, raw material costs, and access to prescription medication, could cause a significant impact to a high proportion of the population.

Recently, a study published by University College London (UCL) (Moncrieff et al., 2022) stated that “after decades of study, there remains no clear evidence that serotonin levels or serotonin activity are responsible for depression.” Furthermore, the report also concluded that anti-depressant drugs worked only a little better than placebo. Moreover, the group of patients that do respond to anti-depressant medications, are not easily identified by clinicians prior to prescribing. In addition, research conducted by drug companies on anti-depressant medication is primarily short-term, so little is known about how well patients do after a few months. Furthermore, when is the right time to taper the patients’ medication with the view of stopping the anti-anxiety and/or anti-depressant drug (s) (Avon and Wiltshire, 2016; Chaplin et al., 2023)? Of primary concern is the numerous side-effects that patients may experience when taking anti-depressants and trying to reduce the medication with the goal of cessation e.g., agitation, unsteadiness, nausea and vomiting, indigestion and stomach aches (including heartburn), diarrhoea and/or constipation, loss of appetite, weight gain and weight loss, headaches, suicidal ideation, emotional numbing, insomnia (problems falling asleep and problems staying asleep), dysphoria, and sexual dysfunction, to name but a few (Braund et al., 2021; Medical news today, 2023).

It is concerning that one in four of our female population is currently medicated with an anti-depressant. How do we change the prescribing culture in Northern Ireland and what safeguards are in place to offer alternative therapies for those seeking help for mental health issues who do not want to be medicated? There are many questions with no answers to the issues raised including; what impact do these drugs have on our physiological, psychological and psychiatric wellbeing? What are the long-term implications of taking these medication(s) on our physical health and are there any links to cognitive impairment (e.g., dementia, Alzheimer’s disease) (Gray and Hanlon, 2018; Richardson et al., 2018; Zheng et al., 2021)?—these questions require urgent investigation and review.

The aim of the current study was to investigate the prescribing culture for anti-anxiety and anti-depressant medication across local government districts in Northern Ireland, which currently holds the unenviable record (in the world) for prescribing the most anti-depressants per head of population (BBC News, 2023a). The study also reviewed the prescribing culture for anti-anxiety and anti-depressant medication across the sociodemographic landscape of Northern Ireland. This information will further highlight the growing trend in our prescribing culture and identify geographical hot spots where resources could be deployed to improve prescribing and the deprescribing culture in Northern Ireland.

## Methods

### Prescription data

Prescription data was obtained from the Northern Ireland Statistics and Research Agency (NISRA) (www.nisra.gov.uk/ninis) from January 2022 to December 2022 (GP Prescribing Data, 2023). The data covers prescriptions in Northern Ireland issued by GPs or Nurses (within a GP Practice), that are subsequently dispensed by a community pharmacist, dispensing doctor, or appliance supplier, and are finally submitted to the Business Services Organisation (BSO) for payment (and have been paid). All dataset records (metadata) are published on opendatani.gov.uk (Opendata Ni, 2023) and are licensed under an Open Government Licence v3.0 (Supplementary S1).

### British national formulary code

Prescriptions of all medications for mood and anxiety under investigation are coded 4.1.2 and 4.3 in the British National Formulary (BNF) (OpenPrescribing, 2023).

### GP practice data

Northern Ireland GP practice location and size data was obtained from NISRA’s quarterly reference file of active GP practices and their list size for October 2022 (GP Practice List, 2023).

### Anti-depressant medication data

The percentage of the population in Northern Ireland receiving anti-depressants by age and gender was obtained from NISRA’s—Family Practitioner Services, General Pharmaceutical Services, Annual Statistics Reports 2020/2021 (published in June 2021) (Family Practitioner Services General Pharmaceutical Services, 2020) and 2021/2022 (published in June 2022) (Family Practitioner Services General Pharmaceutical Services, 2021).

All content is freely available and reproducible under an Open Government Licence v3.0. Data was collected on prescription medications, prescription by age, gender, district, and socioeconomic class.

### Postcode and index of multiple deprivation data

Northern Ireland postcode and Index of Multiple Deprivation (IMD) data was obtained from doogal.co.uk (Doogal, 2023). A mean IMD for each constituency was taken as an estimate of the overall IMD for each constituency. The IMD was based on income (22.5%), employment (22.5%), education (13.5%), health (13.5%), crime (9.3%), barriers to houses and services (9.3%), and living environment (9.3%).

### Northern Ireland parliamentary constituency boundary data

Northern Ireland Parliamentary constituency boundary data was obtained from https://www.opendatani.gov.uk/ (Parliamentary Constituencies, 2023).

### Data analysis

Prescription and practice data was joined by practice number; postcode area data was then joined by practice postcode. The data was grouped by parliamentary constituency and the sum of total items prescribed, and the sum of registered patients was calculated together with the mean of the IMD. The total number of items prescribed per registered patient was then calculated. The data obtained was merged, cleaned and filtered using R (R Core Team, 2021) statistical programming language. Further comparisons and visualisations were generated using R.

## Results

### Demographic

**FIGURE 1:**
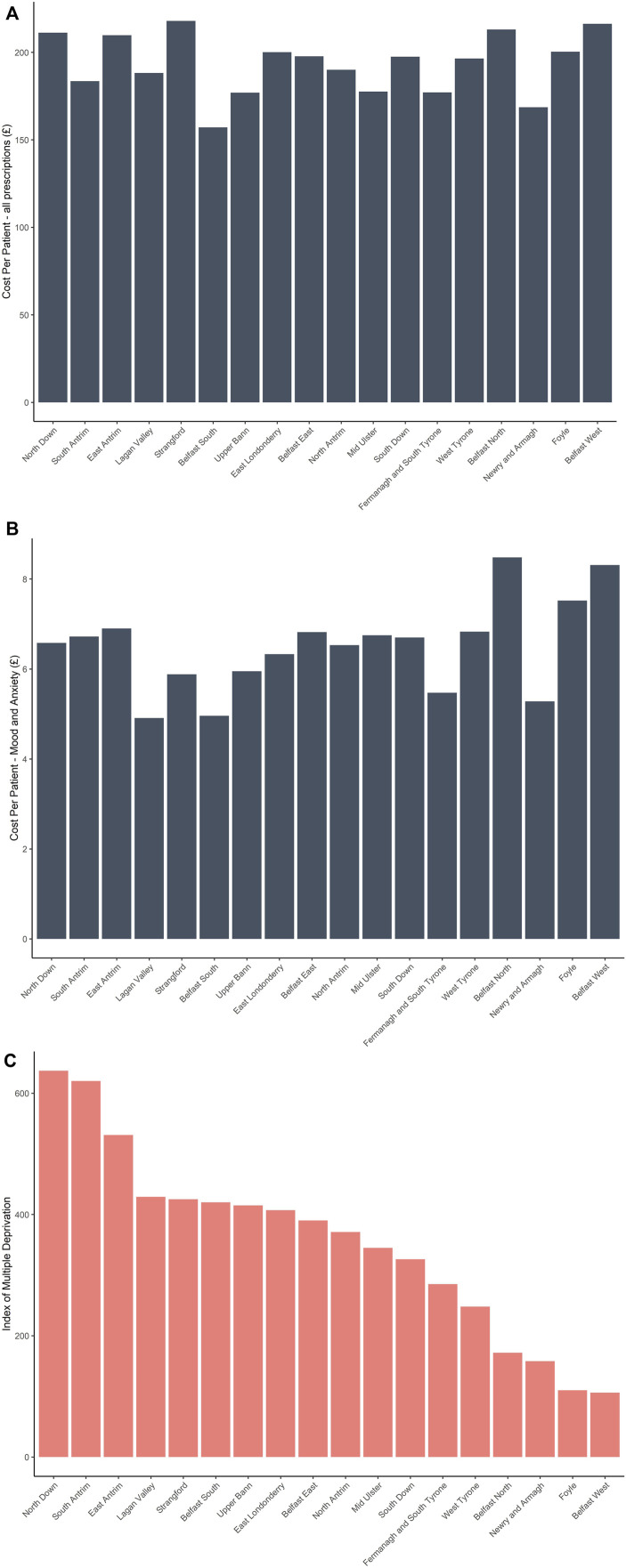
Cost per patient (£)—all prescriptions dispensed in Northern Ireland across all constituencies **(A)**; cost per patient (£)—mood and anxiety prescriptions in Northern Ireland across all constituencies **(B)**; and Index of multiple deprivation across all constituencies in Northern Ireland **(C)**.

The population of Northern Ireland in 2021 was 1,903,100 (50.8% female) (UK Population Data, 2023). To determine the geographical spread of all prescribed medications in Northern Ireland, the data was analysed to show the actual cost of all medications per patient across the different parliamentary constituencies (Figure 1A). The constituencies (North Down, South Antrim, East Antrim, Lagan Valley, Strangford, Belfast South, Upper Bann, East Londonderry, Belfast East, North Antrim, Mid Ulster, South Down, Fermanagh and South Tyrone, West Tyrone, Belfast North, Newry and Armagh, Foyle, and Belfast West) were chosen because this information was common across all datasets. Patients in North Down, East Antrim, Strangford, Belfast North, and Belfast West had more money spent on them than in any other constituencies [actual cost per patient (£)]. Mood and anxiety prescriptions (cost per patient) were highest for Belfast North, Belfast West and Foyle (Figure 1B). Unsurprisingly, the number of mood and anxiety prescriptions were highest in areas with a low index of multiple deprivation (Figure 1C) (higher unemployment, higher debt, and limited access to health resources). Furthermore, living in areas of low socio-economic status can expose individuals to higher stressors, such as unsafe and noisy environments, which can severely impact mental health.

### Prescribing culture for anti-depressant medication in Northern Ireland (2020–2022)

The number of individuals (both male and female) prescribed anti-depressant(s) medication in Northern Ireland marginally increased between 2020/2021–2021/2022; Table 1 (2020/2021) and Table 2 (2021/2022). In 2020/2021 and 2021/2022 females in Northern Ireland were prescribed, almost twice as many, mood and anxiety medications compared to males.

**TABLE 1:** Individuals receiving anti-depressant (s) medications by gender (2020/2021).

Gender	Number of individuals	Percentage (%)
Male	129,694	36.3
Female	227,428	63.7
Northern Ireland	357,122	100

Source: FPS, General Pharmaceutical Services Annual Report 2020/2021.

**TABLE 2:** Individuals receiving anti-depressant (s) medications by gender (2021/2022).

Gender	Number of individuals	Percentage (%)
Male	135,471	36.4
Female	236,663	63.6
Northern Ireland	372,134	100

Source: FPS, General Pharmaceutical Services Annual Report 2021/2022.

**FIGURE 2:**
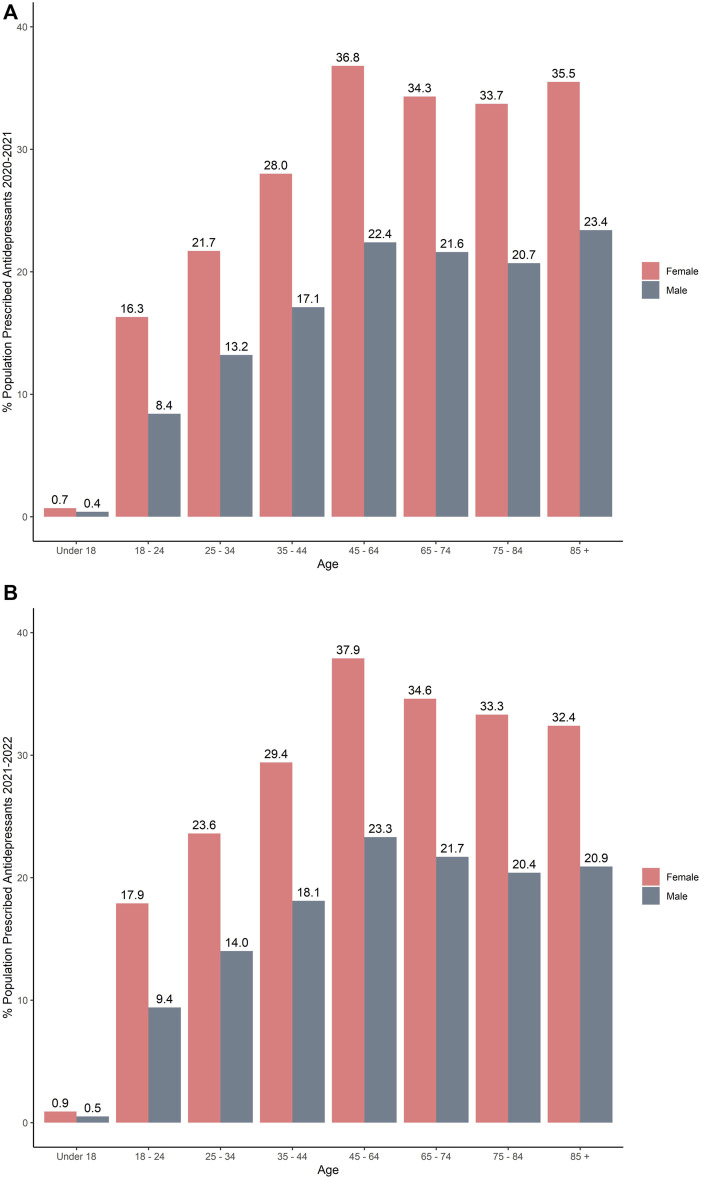
Percentage of the population in Northern Ireland receiving anti-depressant (s) medication by age and gender: **(A)** 2020/2021; **(B)** 2021/2022.

Interestingly, of all the females being prescribed anti-depressant medications, females in the 45–64 age category were prescribed more anti-depressant prescriptions than any other age group between 2020/2021 and 2021/2022 [Figure 2A (2020/21) and Figure 2B (2021/22)].

### Mood and anxiety items prescribed

According to NISRA prescription data from January 2022 to December 2022, a total of 4,483,024 mood and anxiety medications were prescribed to patients across Northern Ireland (Table 3). Of these, serotonin selective reuptake inhibitors (SSRIs) were the most commonly prescribed group of mood and anxiety medications with a total of 1,984,096 (44.3%) items being prescribed. Sertraline was the most commonly prescribed SSRI (46.2%), followed by citalopram (26.6%). Amitriptyline was the most commonly prescribed tricyclic medication and represented 88.7% of this class of drug given to patients across Northern Ireland.

**TABLE 3:** Mood and anxiety medication, according to the BNF, prescribed in Northern Ireland between January 2022 to December 2022.

BNF	Medication	Total No of items prescribed in Jan-December 2022 in NI	Actual cost (£)
**BNF 4.1.2 Anxiolytics**	Oxazepam	2,579	14,797.57
	Chlordiazepoxide	6,793	46,339.97
	Lorazepam	28,860	296,229.54
	Buspirone	83,694	1,014,973.85
	Diazepam	576,185	1,156,509.57
		698,111	2,528,850.50
**BNF 4.3.1 Tricyclics**	Mianserin	118	4,868.25
	Trimipramine	534	297,079.60
	Doxepin	920	199,527.30
	Imipramine	3,559	24,060.52
	Lofepramine	4,410	91,370.54
	Clomipramine	8,227	155,036.19
	Dosulepin	15,628	188,575.64
	Nortriptyline	20,958	77,077.66
	Trazodone	23,320	80,611.19
	Amitriptyline	611,630	829,640.01
		689,304	1,947,846.91
**BNF 4.3.2 MAOIS**	Isocarboxazid	3	204.62
	Phenelzine	75	166,907.15
	Tranylcypromine	92	11,286.00
	Moclobemide	550	22,406.72
		720	200,804.49
**BNF 4.3.3 SSRIs**	Fluvoxamine	1,003	22,542.75
	Paroxetine	38,600	112,035.82
	Escitalopram	91,772	172,117.87
	Fluoxetine	408,418	1,044,687.53
	Citalopram	527,993	697,778.64
	Sertraline	916,310	1,293,432.08
		1,984,096	3,342,594.69
**BNF 4.3.4 Other**	Reboxetine	1,172	23,555.90
	Flupentixol dihydrochloride	1,454	4,343.61
	Agomelatine	3,946	116,951.76
	Vortioxetine	27,434	717,506.53
	Duloxetine	195,471	744,341.54
	Venlafaxine	425,849	2,951,608.07
	Mirtazapine	455,467	621,971.28
		1,110,793	5,180,278.69
	Grand total	**4,483,024**	**13,200,375.27**

The values in bold indicate the total number and the actual cost of mood and anxiety medication prescribed in Northern Ireland between January and December 2022.

## Discussion

Anti-depressants, such as sertraline and citalopram, are selective serotonin reuptake inhibitors (SSRIs) that are prescribed for the treatment of major depressive disorder, anxiety disorders, panic disorder, PTSD, and obsessive-compulsive disorders (Locher et al., 2017). For the past decade, anti-depressants have been among the drugs that bear the so-called “black-box” warning, cautioning GPs that these drugs may increase the risk of suicidal ideation and behaviours in children and young adults (Friedman, 2014).

The serotonin [5-hydroxytryptamine (5-HT)] theory of depression was first postulated in the early 1960 s (Coppen, 1967). However, pharma companies marketed SSRIs for depression and sold the idea that depression was the deeper illness behind the superficial manifestations of anxiety (Healy, 2015). Originally, it was thought that anti-depressants act by increasing the neurotransmission of serotonin, norepinephrine and/or dopamine by blocking one, or several of the reuptake pumps, or their receptors. Deficient levels of these monoamines were thought to cause depression (Delgado, 2000). Therefore, release of more serotonin in several pathways within the brain could, hypothetically, bring about the therapeutic actions of SSRIs in several disorders including obsessive compulsive disorder, panic disorders, bulimia, and binge eating disorders. However, the literature lacks consistent evidence demonstrating a strong link between serotonin and depression. Moreover, there is now an increasing body of research suggesting that there is no convincing evidence that depression, is associated with, or caused by, lower serotonin concentrations or activity. Furthermore, the belief that depression is caused by a chemical imbalance, is now the subject of much debate (Moncrieff et al., 2022). Moreover, without more evidence to the contrary, there is little prospect of agreement in the scientific community any time soon. However, it is generally agreed that SSRIs can be effective in the treatment of anxiety and depression, albeit there is no clear understanding why they work (Kotapati et al., 2019). Most worrying of all is that doctors have no idea what anti-depressants are doing to the brain, long-term, or indeed why they work. Moreover, it is estimated that 10%–30% of patients with major depressive disorder do not respond to typical anti-depressant medications (Rafeyan et al., 2020). However, mental health experts agree that treatment-resistance depression should only be diagnosed in patients who have not been helped by at least two or more anti-depressant treatments (Al-Harbi, 2012). Unfortunately, there is no one size fits all. Furthermore, a standard dose of anti-depressant medication is prescribed independent of gender or BMI. Thus, there is the potential for cumulative toxicity in patients taking more than one medication e.g., statin, anti-hypertensive, pain medication, anti-coagulant, contraceptive, β-blocker (propranolol), for anxiety. In addition, the synergistic interaction of multiple medications (or polypharmacy) is unknown and poorly understood (Brooks et al., 2022). Excessive use of medications has been linked to an increase in falls and cognitive impairment. Furthermore, adverse drug interactions have been estimated as the 4th leading cause of death in US hospitals and are responsible for more than 700,000 hospital visits each year (Malki and Pearson, 2019).

Our health service is stretched to breaking point, compounded by staff shortages [40,000 nurses resigned in 2021 (Independentnurse, 2023)], budget cuts and the immense pressure of dealing with the recent COVID-19 pandemic. Furthermore, new research has suggested that the NHS now faces the impossible task of tackling a rising demand for mental health services (2022). Therefore, it is not surprising that the number of prescriptions for anti-depressants in the UK doubled between 2007 and 2017, from almost 40 million to more than 82 million (Iacobucci, 2019). However, the new National Institute for Health and Care Excellence (NICE) draft guidance (2021) has now recommended that people in England with mild depression should be offered behavioural therapy and/or group exercise, mindfulness and meditation, as possible alternatives to anti-depressant medication (NICE, 2023). However, the PPR Group highlighted concerns that Northern Ireland operates a “postcode lottery” for people who need access to this type of counselling. One in five people in Northern Ireland will experience potential mental health problems in their lifetime (McCool et al., 2022). Therefore, where and when will these new behavioural therapies be available, and more importantly, do we have the infrastructure to support the current NICE guidelines? A holistic approach that provides additional support that assesses the whole individual, not just their mental health issues, and a support structure to address their physical, emotional, social, and spiritual wellbeing, is also required. By providing this type of support network, potentially alleviating some of the stressors, could result in a decrease in the need for pharmacological intervention.

Females are more likely to experience depression than men by a factor of almost 2:1 (Parker and Brotchie, 2010; Shi et al., 2021). Women generally present with more severe symptoms, present at a younger age and tend to experience more prolonged or recurrent depression compared to depressed males (Gulland, 2016; Van Droogenbroeck et al., 2018). The reason behind the disparity between females and males is unknown but may be linked to female hormone levels; higher concentrations of synaptic dopamine in the corpus striatum; and age, causing a greater decrease of synaptic dopamine levels in males than women. In the current study, females in the 45–64 age category were prescribed more mood and anxiety medications than the other age groups. Interestingly, a study in Sweden, reported that men may be undertreated, and women may be overtreated for depression (Sundbom et al., 2017).

Unfortunately, anti-depressant prescription rates continue to rise year on year (England—83.4 million anti-depressant drug items were prescribed in 2021/22—a 5.07% increase from 2020/21). An estimated 8.32 million identified patients received an anti-depressant drug medication in 2021/22—a 5.72% increase from 2020/21 (NHSBSA, 2023; Pulse Today, 2023). Both the number of medications issued and patients receiving anti-depressants have increased for the 6th consecutive year. Do GPs have no other option than to prescribe anti-anxiety and/or anti-depressants?

Recently, a patent application that demonstrated a relationship between SSRI use and elevated serum glial fibrillary acidic protein (GFAP), a potential biomarker of drug-induced cellular toxicity, was published (G01N33/5014, 2020). In the application, the patients prescribed an SSRI medication had significantly higher levels of serum GFAP, when compared to patients not taking anti-depressant medications. Would this suggest that SSRIs can induce cellular toxicity? Most worryingly, an article published in the BMJ (2018) demonstrated that the anti-depressant medications amitriptyline, doseulepin, and paroxetine were consistently associated with incident dementia; this affect was not attenuated by controlling for depression (Richardson et al., 2018). In Northern Ireland, 611,630 amitriptyline, 15,628 doseulepin, and 38,600 paroxetine doses of these medications were prescribed between January 2022 to December 2022. Thus, there is an urgent need for further studies to investigate the potential relationship between SSRI use, serum GFAP and other potential links to incident dementia. Most worryingly, are serum GFAP levels an indicator of neuroinflammatory and/or neurodegenerative diseases, or are serum GFAP levels associated with drug-induced (SSRIs) cellular toxicity (Abdelhak et al., 2022)?

Longitudinal studies investigating relationships between SSRIs and a possible link to a neurodegenerative pathophysiology (cognitive impairment) are urgently warranted to investigate these claims.

## Conclusion

There is a need to collect more data on when and why patients are prescribed an anti-anxiety and/or anti-depressant medication in Northern Ireland. GPs need to be given the time to have regular reviews on anti-depressant use and training on best practice for prescribing (and deprescribing) and managing these classes of drugs. Furthermore, more support for patients affected by severe depression needs to be made available i.e., talking therapies, as indicated earlier. In addition, support services also need to be available for patients affected by anti-anxiety/anti-depressant withdrawal. In addition, there is a need for more research into the benefits and harms of long-term use of these classes of drugs. Finally, there is a need to identify patient populations that would benefit from this class of medication, and the 10%–30% of non-responders, to understand the pharmacogenetics. Moreover, the choice and dose of drug should be titrated based on individual presentation e.g., BMI.

### Limitations of the study

The catchment area for a GP practice may extend beyond the constituency identified and used to present the findings. In addition, the data was obtained from sites identified in the Supplementary Material and is assumed to be reflective and current. Medications prescribed by GPs may be “off-label” (Jannini et al., 2022).
