# Supplementary material for: A review of the prescribing culture of anti-depressants across government districts in Northern Ireland
Source: Front Drug Saf Regul. 2023 Dec 21;3:1303572. doi: 10.3389/fdsfr.2023.1303572 (PMC12443121; doi:10.3389/fdsfr.2023.1303572)
Supplement: Supplementary file 1 [file Table4.DOCX]

**Supplementary 1**

**Data Sources**

[Frequently asked questions - NINIS: Northern Ireland Neighbourhood Information Service (nisra.gov.uk)](https://www.ninis2.nisra.gov.uk/public/StaticFrequentlyAskedQuestions.htm)

The material featured on the NINIS site is subject to Crown copyright protection unless otherwise indicated. The Crown copyright protected material (other than departmental or agency logos) may be reproduced free of charge in any format or medium, under the terms of the Open Government Licence v3.0

For more information we suggest that individuals should visit [Open Government Licence for public sector information](http://www.nationalarchives.gov.uk/doc/open-government-licence).

The material has been reproduced accurately and not used in a misleading context. Where any of the Crown copyright items on this site are being republished or copied to others, the source of the material must be identified, and the copyright status acknowledged.

Source: Neighbourhood Statistics (NISRA) Website: [www.nisra.gov.uk/ninis](http://www.ninis2.nisra.gov.uk/)

Any enquiries regarding the use and re-use of this information resource should be sent to [psi@nationalarchives.gsi.gov.uk](mailto:psi@nationalarchives.gsi.gov.uk).

Users are encouraged to establish hypertext links to this website. The permission to reproduce Crown protected material does not extend to any material on this site which is identified as being the copyright of a third party. Authorisation to reproduce such material must be obtained from the copyright holders.
